# Serum matrix metalloproteinase-9 levels and severity of symptoms in boys with attention deficit hyperactivity disorder ADHD/hyperkinetic disorder HKD

**DOI:** 10.1007/s00787-014-0533-z

**Published:** 2014-03-17

**Authors:** Halina Kadziela-Olech, Piotr Cichocki, Justyna Chwiesko, Jerzy Konstantynowicz, Jan Józef Braszko

**Affiliations:** Medical University, Bialystok, Podlaskie Poland

**Keywords:** ADHD, HKD, Cognitive, Behaviours, MMP-9, Boys

## Abstract

The serum levels of matrix metalloproteinase-9 (MMP-9) in neuropsychiatric disorders of adults have been widely investigated. So far, no studies have been conducted on the relationship of MMP-9 and cognitive domains in children with two phenotype models, attention deficit/hyperactivity disorder and hyperkinetic disorder (ADHD/HKD). The aim of this research was to evaluate and test the hypothesis that serum MMP-9 levels are associated with the severity of symptoms in children with ADHD/HKD and to compare the results in two models of this disorder. The study group comprised 37 Caucasian boys aged 7–12 years with HKD, being a subset of the combined ADHD subtype. Intellectual functions were measured using Wechsler Intelligence Scale for Children-Revised. The analysis of serum concentrations of MMP-9 was based on a quantitative sandwich ELISA. The statistical regression analysis revealed a correlation between increased serum MMP-9 levels and severity of symptoms in the ADHD (*β* = 0.33; *p* = 0.043) and HKD (*β* = 0.34, *p* = 0.037) model. According to the results, elevated levels of serum MMP-9 in boys with HKD may be associated with clinical impulsivity domain (*β* = 0.38; *p* = 0.019).

## Introduction

Matrix metalloproteinases (MMPs) (zinc-dependent proteinases) play important roles in extracellular matrix (ECM) remodelling in physiological and pathological processes [1–6]. The ECM is not only a mechanical support for its constituent cells, but also participates in the regulation of metabolism and in the exchange of information with the environment. To accomplish these functions, the matrix should undergo constant transformation. The imbalance between decay and renewal of ECM is characteristic of most pathologies as their primary or secondary cause [7, 8]. Matrix metalloproteinases are not only able to cleave all the constituents of ECM proteins, but also to activate or deactivate other numerous “signalling” molecules, such as receptors, adhesion molecules and growth factors [1, 5, 9, 10].

Matrix metalloproteinase-9 (MMP-9), a typical ECM protease (gelatinase), is characterized by a wide range of substrates in vitro [4, 6]. Recently, a convincing support for the involvement of MMP-9 in inflammation, autoimmune diseases, cancer metastases and physical injuries has been reported [5]. Recent studies have also focused on the relationship of MMP-9 with the neurological disorders in adults, and its possible role in the reorganisation of ECM after injuries of the nervous system. This gelatinase plays an important role in system dysfunction of the blood–brain barrier [11–15], and its level can be assessed in the plasma [17–19]. The serum levels of MMP-9 in neuropsychiatric disorders in adults have been widely studied. The data focus an potential association of MMP-9 with depression, schizophrenia and bipolar disorder in adults [20]. The results of experiments using rodent models showed a role of MMP-9 in epileptogenesis [21]. Many studies indicate that cognitive dysfunctions may be associated with changes in MMP-9 activity [11, 17, 22–24], and show the importance of MMP-9 in cognitive dysfunctions of adult patients with dementia [19, 25], cerebral ischemia [11, 13, 14, 17] or neuropsychiatric symptoms in autoimmune diseases [16]. So far, no studies have been conducted on the relationship of MMP-9 and cognitive function in children with attention deficit/hyperactivity disorder and hyperkinetic disorder (ADHD/HKD).

Cognitive deficits and behavioural symptoms in ADHD have been studied intensely and are well documented [26–34]. They are classified according to DSM-IVR [35] and DSM-V [36], or ICD-10 [37]. These procedural approaches to both classifications show some differences. The cognitive symptoms of ADHD cover two domains: inattention and hyperactivity/impulsivity. In the version of DSM-IVR or DSM-V, three subtypes have been proposed and evaluated: ADHD predominately inattentive, predominately hyperactive/impulsive (if symptoms involve only one domain) or combined. According to ICD-10, HKD can be diagnosed when symptoms belonging to all the domains are observed, including inattention, hyperactivity and impulsivity.

Neuropsychological studies concerning ADHD show a variety of cognitive deficits, altered level of motivational processes and irregularities in the stages of processing and storage of information in the brain [30–33, 38–40]. Intellectual and neuropsychological abilities in ADHD have been investigated for many years [41–43]. Children with ADHD exhibit a wide range of performance deficits across a range of neuropsychological domains, including response inhibition, working memory, planning, sense of time, sustained attention, and Verbal learning [28, 31–33, 43–45]. Many studies have attempted to determine the characteristic profiles of the intellectual capacity of children with ADHD [27, 42, 46, 47]. Children with ADHD have a lower overall level of intellectual ability than their healthy peers [27, 32, 43, 47], but a similar decrease is also observed in children with other neuropsychiatric disorders [42, 46]. Several studies also point to a difference between Verbal and performance IQ in children with ADHD as compared with healthy children [27, 40, 46, 47], whereas others provide contradictory results regarding these cognitive aspects [40, 42, 48]. Research to date could not fully explain neuropsychiatric deficits in the functioning of children with ADHD and their response to treatment. Differences in the research paradigms along with the heterogeneity of the disease phenotypes are, at least partly, responsible for the problem.

Despite of intense research in recent decades, the brain processes and mechanisms which underlie the cognitive deficits of ADHD are unclear. It can be assumed, that the reorganization of the brain responsible for behavioural and cognitive symptoms of ADHD/HKD, and changes in the activity of MMP-9 may be the result of ECM reconfiguration in this disorder. To date, serum MMP-9 levels in children with ADHD/HKD have not been studied. Therefore, the aim of this study was to evaluate associations between serum MMP-9 levels and the symptoms severity in children with ADHD/HKD. We hypothesized that the activity of MMP-9 may be related to intensity of symptoms and the degree of cognitive dysfunction in children with ADHD/HKD.

## Methods

### Participants and procedure

The study group comprised 37 Caucasian boys with Combined ADHD subtype and HKD, aged between 7 and 12 years (median 9.2 years; 25th percentile—7 and 75th percentile 11 years). The study group was selected among patients with hyperactivity symptoms from primary care, who had been referred to the specialized psychiatric diagnosis and therapy at the Child and Adolescent Psychiatry Unit of the University Children’s Hospital in Bialystok (Poland).

The presence of several inattentive or hyperactive, impulsive symptoms in two or more situation (at home, school, in other activities) is required for the diagnosis of ADHD/HKD [35–37]. Attention deficit/hyperactivity disorder symptoms at study baseline were measured using the NICHQ Vanderbilt Assessment Scale for Parent (VADPRS) and for Teacher (VADTRS) [49], each of which being divided into two sections: symptoms and performance. The VADPRS contains 47 items, and VADTRS includes 35 items of symptoms and 8 items of performance. The construction of the toolkit is based on the Diagnostic and Statistical Manual of Mental Disorders, Fourth Edition (DSM-IV) [35], and includes the 18 ADHD items (9—inattention and 9—hyperactive/impulsive symptoms), and as well as a screen for the following coexisting conditions: oppositional-defiant disorder, conduct disorder, anxiety and depression. The 4-point Likert scale rates the severity of symptoms (i.e., 0 = never, 1 = occasionally, 2 = often, and 3 = very often), whereas the 5-point Likert scale assesses performance (i.e., 1 = excellent, 2 = above average, 3 = average, 4 = somewhat of a problem, 5 = problematic). For Combined Inattention/Hyperactivity subtype, at least six of nine items of inattention and at least six of nine items of hyperactive/impulsivity need to score two or three points, with at least one of performance questions scoring four or five points. The internal consistency and structure of the Vanderbilt Assessment Scales are acceptable and consistent with DSM-IV and other accepted measures of ADHD [50, 51]. This toolkit has recommendations of American Academy of Pediatrics as a framework for diagnostic decision making in a child 6–12 years old [49].

Both classifications were used to identify a homogeneous phenotype of HKD/ADHD. The diagnosis of Combined ADHD subtype was ascertained using current DSM-IV criteria [35], stating that one must have at least six positive responses to either the inattentive nine and six out of nine items on hyperactive/impulsive nine core symptoms. The diagnosis of HKD was performed according to the criteria of ICD-10 [37], requiring symptoms of three domains (at least six of nine items of inattention, at least three of five items of hyperactive and at least one of four of impulsivity). In the study group, these symptoms were present before the child reached 7 years of age, in a number of situations, continued on regular basis for more than 6 months and significantly impaired the child’s academic and social functioning. For the study, the 18 ADHD items were examined by separating symptoms into subscales of inattention, hyperactivity and impulsivity. Mean (SD) subscale scores were computed across each rate for each symptom domain.

Each examination comprised observation of the family and other informants, attention being paid to parents/guardians personality traits and attitudes to the child, relationships of parents, the child’s behaviour towards his/her parents/guardians, the child’s behaviour and spontaneous play and group functioning. Exclusion criteria of the study were the coexistence of other psychiatric or neurodevelopmental disorders (e.g. autistic spectrum, obsessive–compulsive, oppositional-defiant, conduct disorders, anxiety, depression, tics), epilepsy, mental retardation and somatic diseases. None of these boys had history of pharmacological treatment.

All participants in the study were administered the Wechsler Intelligence Scale for Children-Revised (WISC-R, Polish adaptation) [52]. The intelligence quotient was evaluated by a certified psychologist. Wechsler Intelligence Scale for Children-Revised includes Verbal (subtests: general information, similarities, arithmetic, Vocabulary, understanding) and performance (subtests: picture completion, picture arrangement, block design, object assembly, coding-digit symbol) scales. Raw scores obtained in subtests results were restated for conversion in accordance with the standards of the corresponding age group. The sum of the results converted from individual subtests create an overall score for the Verbal and performance scales. The values corresponded to the amount of IQ results translated into Verbal scales, wordless, and full of different age groups. In addition to the standard deviation scores of these subtests, Verbal intelligence scale, performance scale, and full-scale IQ coefficients were calculated. The mean value for all intelligence scales was 100 and the standard deviation 15. The diagnosis of children was confirmed by certified psychiatrist and psychologist.

Venous blood samples of each child with ADHD were taken during the run psychiatric and psychological diagnosis. After centrifugation, the serum was frozen and stored at −70 °C until the time of the signs. Matrix metalloproteinase-9 activity in the serum was determined by ELISA, using a kit Human ELISA System Biotrak (GE Healthcare, Amersham Biosciences) and expressed in μg/l. To minimize assay variance, serum levels of MMP-9 from each subject were measured on the same day. The assay was based on a two-site ELISA sandwich format using two antibodies directed against different epitopes of MMP-9. Protocols were performed according to the manufacturer’s instructions. The sensitivity defined as two standard deviations above the zero dose binding was determined as 0.6 μg/l, assay range 4–128 μg/l.

### Statistical analysis

All statistical analyses were performed using the Statistica 10.0 PL (StatSoft). Since many variables were not normally distributed according to the Shapiro–Wilk test, the analysis used non-parametric tests: the Kruskal–Wallis test with post hoc test, Mann–Whitney *U* test and Spearman’s rank correlation. The values of the variables are presented as mean ± SD or median, first and third quartile. The regression analysis was performed. The univariate linear regression models were created. The *p* value <0.05 was considered statistically significant.

The study was approved by the Ethical Committee of the Medical University of Bialystok, in accordance with the principles of Guidelines for Good Clinical Practice R-I/003/168.

## Results

Both models (HKD and combined ADHD subtype) were identified in every boy in the study group. Total scores were similar for the clinical model of HKD (mean ± SD: 14.68 ± 2.00; median 14, first and third quartile:14–16) and ADHD (mean ± SD: 14.76 ± 1.62; median 14, first and third quartile:14–16). No significant differences were noted between the average values of VADPRS (mean ± SD: 39.95 ± 6.81) and VADTRS (mean ± SD: 38.14 ± 6.14) in the study group. There were significant positive correlations between ICD-10 and DSM-IV symptoms (*R*_s_ = 0.95; *p* < 0.001) and VADPRS (*R*_s_ = 0.69; *p* = 0.001) (Table 1). In addition, the interrelationship between HKD and ADHD domains was tested. The number of hyperactivity symptoms of HKD correlated significantly with hyperactivity/impulsivity of ADHD (*R*_s_ = 0.76; *p* < 0.001), however the relationship between the impulsivity of HKD and ADHD domain was not significant (*R*_s_ = 0.32; *p* = 0.06). There was a highly significant correlation between inattention of HKD and inattention of ADHD (*R*_s_ = 0.98; *p* < 0.001) (Table 1).

**Table 1:** Cognitive domains and intellectual functions in boys with HKD

	Mean (SD)	HKD model					
		Inattention		Hyperactivity		Impulsivity	
		*R*_s_	p*^,^ **	*R*_s_	p*^,^ **	*R*_s_	p*^,^ **
ICD (total)	14.68 (2.00)	0.82**	<0.001	0.70**	<0.001	0.38*	0.019
Inattention	7.11 (1.17)	1		0.32		0.24	
Impulsivity	3.16 (0.73)	0.24		−0.10		1	
Hyperactivity	4.43 (1.12)	0.32		1		−0.10	
Combined ADHD subtype							
DSM-IV (total)	14.76 (1.62)	0.89**	<0.001	0.56**	<0.001	0.35*	0.03
Inattention	7.03 (1.14)	0.92**	<0.001	0.26		0.27	
Hyperactivity/impulsivity	7.73 (0.87)			0.76**	<0.001	0.32	
VADPRS (total)	39.95 (6.81)	0.73**	<0.001	0.35*	0.03	0.26	
Inattention	19.29 (4.31)	0.74**	<0.001	0.08		0.25	
Hyperactivity/impulsivity	20.68 (3.57)	0.50**	0.001	0.61**	<0.001	0.20	
VADTRS (total)	38.14 (6.14)	0.19		0.05		0.08	
Inattention	19.27 (3.68)	0.23		0.04		0.09	
Hyperactivity/impulsivity	19.03 (3.81)	0.22		0.06		0.10	
Total IQ	98.43 (14.12)	−0.21		0.10		−0.10	
Verbal IQ	99.40 (15.31)	−0.11		0.13		0.02	
Performance IQ	97.32 (14.41)	−0.06		−0.01		−0.30	
Information	10.38 (3.34)	−0.09		0.12		0.15	
Similarities	10.32 (2.68)	−0.01		0.15		0.06	
Arithmetic	8.95 (3.47)	−0.06		−0.04		0.07	
Vocabulary	10.00 (3.59)	−0.42**	0.01	0.21		−0.15	
Comprehension	10.14 (2.18)	−0.49**	0.002	−0.05		−0.22	
Picture completion	10.49 (1.74)	−0.05		−0.10		0.08	
Picture arrangement	10.57 (2.90)	−0.03		0.19		−0.05	
Block design	9.27 (3.39)	0.02		−0.04		−0.28	
Object assembly	9.32 (2.69)	−0.04		0.19		−0.07	
Coding-digit symbol	8.05 (3.06)	−0.31		−0.12		−0.32	

The comparison of DSM-IV to ICD-10. Statistical coherence of values was determined by Spearman’s rank correlation test. The statistical correlation between intellectual functions and domains of HKD was determined by Spearman’s rank correlation test

*R*_s_ Spearman’s rank correlation coefficient

* Significant correlation at the 0.05 level (two-sided)

** Significant correlation at the 0.01 level (two-sided)

The median values of the total IQ-98 (25th–75th percentile: 90–106), the Verbal IQ-102 (25th–75th percentile: 91–108) and the performance IQ-90 (25th–75th percentile: 87–106) were determined in all the study subjects. The Vocabulary subtest and the Comprehension subtest with the inattention domain showed a negatively significant correlation (*R*_s_ = −0.42; *p* = 0.01 and −0.49; *p* = 0.002, respectively) (Table 1).

**Fig. 1:**
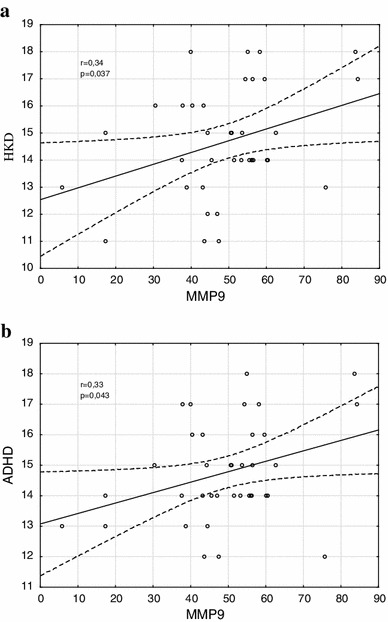
The symptom severity and MMP-9 levels in boys with HKD/ADHD

**Fig. 2:**
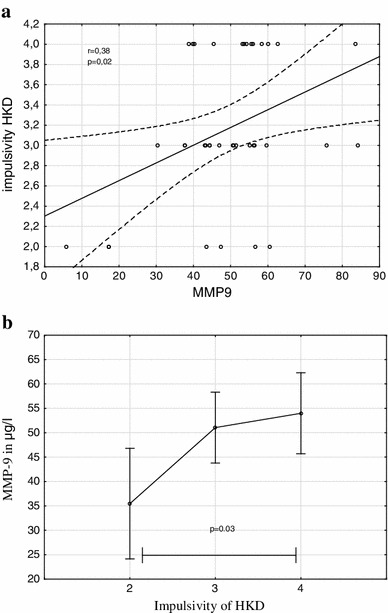
MMP-9 levels and Impulsivity

The mean (SD) of MMP-9 levels 49.13 (15.86) μg/l and median of MMP-9 levels 50.82 μg/l (25th percentile—43.14 μg/l; 75th percentile—56.34 μg/l) in study group amounted. The General Regression Models (GRM) were used to assess the effect of MMP-9 on HKD/ADHD symptoms (Table 2). The MMP-9 levels were significantly associated with symptoms severity of HKD and of ADHD (*β* = 0.34; *p* = 0.037 and *β* = 0.33; *p* = 0.043, respectively) (Fig. 1a, b). Furthermore, serum MMP-9 concentrations correlated with increase impulsiveness (*β* = 0.38; *p* = 0.019) (Fig. 2a, b).

**Table 2:** MMP-9 levels in relation to cognitive domains of HKD/ADHD

	*b*	SE	95 % CI		*β*	*p*
HKD						
MMP-9	0.043	0.02	0.003	0.084	0.34*	0.037*
Inattention						
MMP-9	0.021	0.012	−0.003	0.045	0.29	0.085
Hyperactivity						
MMP-9	0.004	0.012	−0.021	0.028	0.05	0.770
Impulsivity						
MMP-9	0.018	0.007	0.003	0.032	0.38*	0.019*
ADHD						
MMP-9	0.034	0.016	0.001	0.067	0.33*	0.043*
Innatention						
MMP-9	0.021	0.011	−0.002	0.045	0.29	0.075
Hyperactivity/impulsivity						
MMP-9	0.012	0.009	−0.005	0.031	0.24	0.160

*β*-standardized regression coefficient parameterization of sigma limits

*b* regression coefficient, *SE* standard error, *CI* confidence intervals

* Regression model statistically significant *p* < 0.05

Analysis of the relationship between serum activity of MMP-9 and WISC-R subtests quotient measurements revealed no correlation. There was no relationship between age and the level of MMP-9 in the study group.

## Discussion

To our best knowledge, this is the first study to assess the levels of MMP-9 in children with HKD/ADHD, and to investigate the correlation of MMP-9 with the cognitive function and symptoms severity. A certain limitation of our study was small group size. However, our intention was to include children with a very similar phenotype of HKD/ADHD. In our study, the increased serum MMP-9 levels was correlated with the severity of symptoms in the HKD/ADHD clinical model. Based on the data, elevated levels of serum MMP-9 in boys with HKD were specifically associated with clinical impulsivity domain.

The motivation and reward, may represent another core deficit for ADHD [53]. Children with ADHD/HKD are incapable of to the self-control, reveal rapid unplanned reactions and difficulty of postponing award [54, 55]. Experimental models of the impulsivity emphasize repeatability even though behaviours are punishable [56]. The concept of impulsivity domain for HKD in accordance with the ICD-10 includes four symptoms, whereas the impulsivity is not a separate domain for ADHD in DSM IV or DSM V [34–37, 57]. One of the four impulsivity symptoms for HKD: “Talks excessively” is categorized as a symptom of hyperactivity in DSM-IV and DSM-V [36, 37]. This had important implications for the interpretation of our findings. Unclear conceptualization of the impulsivity domain may have influenced the differences in the correlation between serum MMP-9 levels and HKD-model or ADHD-model in the study group.

Our research shows that the severity of symptoms affects WISC-R results. We found a significant negative correlation between severity of inattention and the Vocabulary and Comprehension subtests, which form Verbal Conceptual Thinking (can shape account for social intelligence, verbalization, memory recall) [33, 58]. It has been proved that the attention deficit is most disturbing factor in the social functioning of children with ADHD/HKD. Symptoms of inattention hinder to establish social relationships through observation and focus on the social factors, that are essential for supporting interaction [30, 31, 59]. Most authors agree that children with a diagnosis of combined ADHD subtype present with difficulties in the largest number of areas of cognitive and psychosocial functioning [32, 40, 41, 44, 47], but the reasons for the existence of cognitive deficits in children with ADHD/HKD are still unclear. Recent PET brain imaging studies revealed, that the most DA deficits were evident in the ventral striatum (modulation of reward and motivation) and in the midbrain (where most DA neurons are located) [60], which supports the DA hypothesis of ADHD [61, 62], although the specific details are not yet clear. Dopamine transporter DAT, which is located in dendrites and axonal membrane, or activation of dopamine receptors may be responsible for dopamine levels [62, 63]. Although most studies have been focused on the prefronto-striatal system [64], others, concerned with psychopharmacology and neuroimaging (MRI, fMRI) of brain processes, have pointed to many cortical and subcortical brain regions implicated in ADHD [65–67]. Furthermore, it has been recently shown that many neural networks are involved in cognitive and behavioural symptoms of ADHD [68–71]. Researchers who have defined the pattern for typical brain development suggest that some childhood onset cases may be disorders of neuroplasticity [72–74]. Some studies support this hypothesis for autism or schizophrenia [73, 75]. The plasticity is a property of the nervous system that provides the capability for adaptation, self-healing, learning and memory [73].

The extracellular proteolysis by MMP-9 is important for the functional and structural synaptic plasticity [1, 2, 5–7, 9, 10]. Matrix metalloproteinase-9 is expressed in the hippocampus, striatum, diencephalon, midbrain, cerebellum and frontal cortex of the rat [3, 6, 76, 78], with the greatest activity in the hippocampus [14, 15, 24, 77]. The contribution of MMP-9 in addictions to methamphetamine [79, 80] or cocaine [76] may suggest its role in dopamine neurotransmission. This enzyme, located in dendritic spines is responsible for the structural changes in synapses and thereby may be partly responsible for the improper regulation of extracellular dopamine levels [81]. Matrix metalloproteinase-9 is required for the formation of abnormal synaptic connections between hippocampal granule cell axons and their dendrites in rodent brain, and is also related to immature dendritic spine morphology [82]. The level of this enzyme is elevated in Fragile X syndrome (FXS), in which inattention, impulsivity and hyperactivity are manifested beside autism, and administration of minocycline (MMP-9 inhibitor) to mouse model of FXS results in normalization of behaviours and a decrease in anxiety [83]. Despite the absence of clinical symptoms of ASD in our study boys, according to research, inadequate social behaviours in children with ADHD may be phenomenologically and etiologically related to autism spectrum disorders [84, 85].

The MMP-9 concentration increases in inflammation, hypoxia or injuries of brain, where the blood–brain barrier is damaged [11–17, 86]. This leads to the hypothesis that prenatal environmental risk factors for ADHD, such as viral infections, asphyxia, neurotoxins, alcohol or nicotine can affect the expression of MMP-9, and consequently the long-term alteration in blood–brain barrier permeability to small-molecular-weight markers [12, 75]. Minocycline through inhibition of MMP-9, reduces permeability of sucrose during intracerebral injection [86]. Another hypothesis is related to continuous disturbances in MMP-9 gene transcription by unknown specific factors, which may be supported by the result of mutation in FMR1gene (leading to autism), in which the FMpR protein, natural of MMP-9 translation inhibitor, is missing. In the animal model (Fmfr1KO mice), administration of minocycline resulted in maturation hippocampal dendritic spines and behaviour improvement [83].

Though the relevant scientific studies gained great interest in recent decades, the etiopathogenesis and underlying mechanism of ADHD/HKD remain still unclear. So far, laboratory, psychological or biological studies that could be specific enough to allow explicit in diagnosis of ADHD/HKD have been missing [36]. The structuralised interview and either ICD-10 or DSM-IV/DSM-V are the fundamental elements of diagnosis. However, some variation in the domain of impulsivity may suggest difficulty in understanding the place for cognitive deficits in the clinical diagnosis of ADHD/HKD. Therefore, a genetic defect determines a certain predisposition to ADHD/HKD but environmental factors contribute largely to the phenotype of the disorder [72, 87]. The mechanism of the impact of unexplained environmental factors is not clear, but MMP-9 may be associated with the severity of symptoms.

To understand the processes engaged in cognitive dysfunction in ADHD/HKD, it is necessary to unravel signalling pathways, complex interaction networks and metabolic alterations involving many anatomical components. However, we are aware that the role of MMP-9 in neurodevelopmental damage still remains unclear, and the present study is the first to show the elevated levels of serum MMP-9 in boys with HKD correlated with severity of the disorder. The results indicate that the increased levels of serum MMP-9 in boys with HKD are associated with the clinical domain of impulsivity.
